# CNS Tumor with BCOR/BCORL1 Fusion: A Rare Tumor Entity

**DOI:** 10.3390/ijms26146729

**Published:** 2025-07-14

**Authors:** Jerry Lou, William Yong, Kenneth Aldape, Eleanor Chu, Caressa Hui, Frank P. K. Hsu, Michelle Zheng, Anatevka Ribeiro, Gianna Fote, Daniel Na, Carlen A. Yuen

**Affiliations:** 1 Department of Pathology, University of California Irvine, Irvine, CA 92697, USA; 2 Chao Family Comprehensive Cancer Center, University of California Irvine, Irvine, CA 92697, USA; 3 Laboratory of Pathology, Center for Cancer Research, National Cancer Institute, National Institutes of Health, Bethesda, MD 20892, USA; 4 Department of Radiology, University of California Irvine, Irvine, CA 92697, USA; 5 Department of Radiation Oncology, University of California Irvine, Irvine, CA 92697, USA; 6 Department of Neurosurgery, University of California Irvine, Irvine, CA 92697, USA; 7 UC Irvine Charlie Dunlop School of Biological Sciences, University of California, Irvine, CA 92697, USA; 8 Department of Neurology, Division of Neuro-Oncology, University of California Irvine, Irvine, CA 92697, USA; 9 Department of Research, University of California Irvine, Irvine, CA 92697, USA

**Keywords:** PNET, CNS tumor with BCOR/BCOR(L1) fusion, CNS tumor with BCOR internal tandem duplication, ependymoma, temozolomide

## Abstract

Central nervous system (CNS) tumor with BCL6 corepressor gene BCOR/BCORL1 fusion is an extremely rare tumor entity, with fewer than 40 cases reported. These tumors are distinct from the WHO 2021-defined CNS tumor with BCOR internal tandem duplication. Even rarer are CNS tumors that match to the methylation class of CNS tumors with BCOR/BCORL1 fusion, but lack fusions and instead harbor truncating small nucleotide variants in BCOR. To our knowledge, only two other cases of this scenario have been previously reported. Due to their scarcity and morphological features that mimic oligodendrogliomas and ependymomas, the diagnosis of CNS tumor with BCOR/BCORL1 fusion can be challenging, and misdiagnoses are not uncommon. Histologic findings of Olig2 positivity with focal to absent GFAP warrant further evaluation for this tumor entity. Moreover, no standard of care therapy exists for these tumors, making treatment selection difficult. We present a case of a 37-year-old woman with a midline CNS tumor with BCOR/BCORL1 fusion, harboring a pathogenic BCOR c.626del (p.S209Cfs*7) (Exon 4) variant, who was successfully treated with definitive radiation therapy and adjuvant temozolomide. Notably, EMA showed focal strong dot-like perinuclear immunoreactivity, which has not been previously reported in these tumors. This case adds to the limited but growing body of evidence supporting the use of radiation and temozolomide in treating tumors matching the methylation class of CNS tumors with BCOR/BCORL1 fusion without a detectable fusion.

## 1. Introduction

The World Health Organization (WHO)’s classification of central nervous system (CNS) tumors continues to undergo revisions to improve the diagnostic accuracy for CNS-primitive neuroectodermal tumors (PNETs). Each new iteration refines and expands upon the existing diagnostic categories. The 2021 WHO classification of CNS tumors defined a new CNS-PNET tumor entity: CNS tumor with BCL6 corepressor gene (BCOR) internal tandem duplication (ITD) [1,2,3]. The BCOR gene, located on Xq26.1, is a tumor suppressor gene and an epigenetic regulator that mediates cell differentiation and structural development, while contributing to polycomb repressive complexes (PRCs) [4,5,6,7,8,9,10]. PRC1 is a main regulator of cellular outcome and stem cell existence [10,11]. PRC1.1 alterations are associated with BCOR and BCOR(L1) [10]. Accordingly, a loss of function mutation in the BCOR gene interrupts tumor suppression functioning and thereby leads to tumorigenesis. Additionally, ITDs and oncogenic gene fusions are alternate drivers of tumorigenesis [3,12,13,14].

The updated WHO classification schemes have increasingly incorporated DNA methylation profiling as an ancillary diagnostic tool. A diagnosis of CNS tumor with BCOR-ITD can be designated by histologic criteria coupled with an ITD in Exon 15 of BCOR or DNA methylation profiling in unresolved cases [2]. CNS tumor with a BCOR or BCOR Like 1 (L1) fusion entity has a unique DNA methylation signature that is molecularly distinct from CNS tumor with BCOR-ITD and is not yet recognized in the WHO classification of CNS tumors [15]. BCOR-L1 is a BCOR homolog and is an intense transcriptional corepressor when tethered to a heterologous promoter [9,16]. Limited information exists for this exceptionally rare tumor that is characterized by the presence of a BCOR/BCOR(L1) fusion, copy number variations within chromosomes Xp11.4 and 22q12.31, or truncating small nucleotide variants [14,17]. Moreover, little is known about the relationship between BCOR-fusion tumors and BCOR-ITDs. However, biological differences have been reported between these tumors. In stark contrast to BCOR-ITD tumors, BCOR-fusion tumors more commonly occur in adults [14,18,19]. Evidence is conflicting for any sex differences [14,20]. The median age at presentation for patients with BCOR-fusion tumors is 27–30 years of age, but broadly presents between the ages of 5 and 72 years [14,20]. The median progression-free survival for these tumors is 16 months, but ranges from 1 to 86 months [14,18,19]. The median overall survival is 27 months and data suggest that patients with BCOR-fusion tumors portend better prognosis compared to BCOR-ITD [18,19,20].

On diagnostic imaging, CNS tumors with BCOR/BCOR(L1) fusion commonly arise within or adjacent to the ventricles and are characteristically T1-hypointense and T2-hyperintense [17,19]. These tumors can be well-demarcated or diffusely infiltrating and with or without enhancement [17,18].

CNS tumors with BCOR/BCOR(L1) fusion are mimickers of other diseases. Histology demonstrates intra- and inter-tumor heterogeneity, including oligodendroglioma-like, ependymoma-like, and embryonal features, frequently with focal calcifications and/or myxoid change [14,17]. Tumor cells show diverse cytology ranging from round to ovoid, and occasionally angulated nuclei with delicate to hyperchromatic chromatin and clear to eosinophilic cytoplasm, occasionally with intracytoplasmic vacuolation [14,17]. Malignant features include necrosis and elevated mitotic activity, and less commonly, microvascular proliferation [14,19]. Ependymoma-like perivascular pseudorosettes and/or oligodendroglioma-like chicken-wire vasculature are consistently encountered [14,17,19,21]. Olig2 is commonly expressed, and GFAP may be focally present or absent [14,17,19]. Accordingly, these tumors can be misdiagnosed as supratentorial ependymoma, oligodendroglioma, astrocytoma, glioblastoma, or ganglioglioma [14]. However, molecular advances have improved upon the classification of CNS tumors. The presence of MYCN amplification or fusions in ZFTA or YAP1 is suggestive of ependymoma [1]. The presence of mutations in IDH1, IDH2, 1p/19q, TERT promoter, CIC, FUBP1, and NOTCH1 is suggestive of oligodendroglioma [1].

Positivity for glial markers in the presence of a BCOR fusion justifies a provisional designation as glioma with BCOR fusion [14]. BCOR fusion or a matching methylation class distinguishes these CNS tumors from BCOR-ITD [14]. BCOR-fusion methylation class tumors are characterized by EP300 or CREBBP as BCOR/BCOR(L1)-fusion partners, an MEAF6::CXXC5 fusion, a BCOR stop mutation, or CNV breakpoints at the EP300 and BCOR loci on chromosomes X or 22 [14,17].

There are fewer than 40 reported cases, warranting further investigations into the clinical behavior and radiographic, pathologic, and genomic features of this rare tumor entity [17]. No standard of care therapy exists, further highlighting the need to deepen our understanding of CNS tumors with BCOR/BCOR(L1) fusion. Herein, we present a case of a 37-year-old female with a CNS tumor harboring a pathologic BCOR c.626del (p.S209Cfs*7) (Exon 4) variant that matched the same methylation class as CNS tumors with BCOR/BCOR(L1) fusion, but without a detectable fusion, who was successfully treated with radiation followed by adjuvant temozolomide.

## 2. Case Presentation

**Figure 1:**
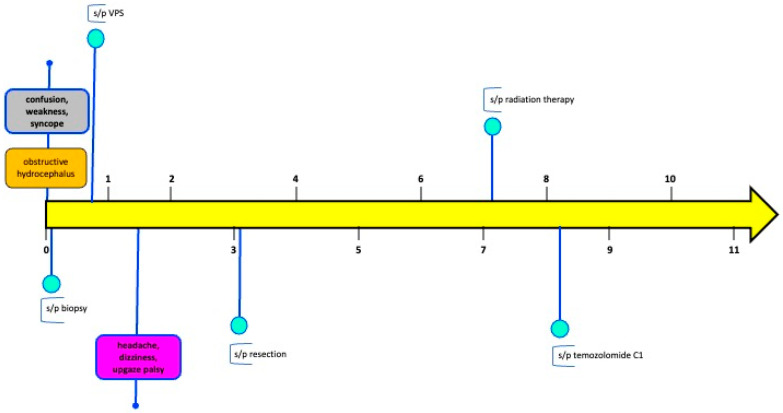
Patient timeline (months).

**Figure 2:**
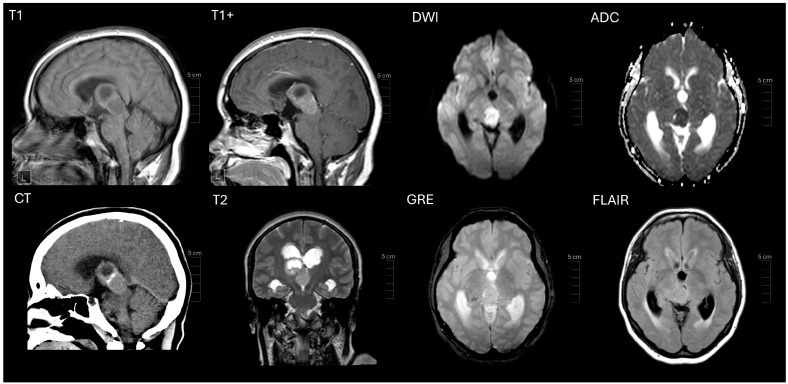
Initial imaging demonstrating an enhancing (T1 and T1+), diffusion-restricting (DWI and ADC), partially calcified (CT), partially cystic (CT), T2-intermediate (T2), tectal-intraventricular mass causing obstructive hydrocephalus (GRE and FLAIR).

**Figure 3:**
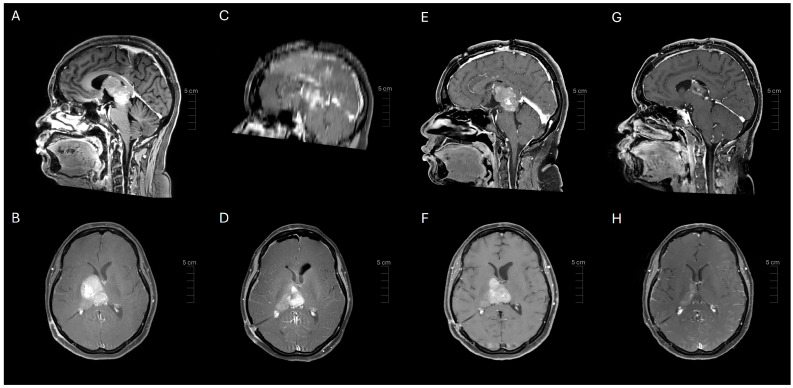
Serial post-contrast imaging demonstrating tumor growth and ventricular shunting (**A**,**B**), tumor debulking (**C**,**D**), continued tumor growth (**E**,**F**), and response to radiation treatment (**G**,**H**).

A 37-year-old female presented to an outside institution with syncope, altered mental status, and visual disturbance (Figure 1). Magnetic resonance imaging (MRI) and computed tomography (CT) demonstrated a 4.3 cm enhancing, diffusion restricting, partially calcified, partially cystic mass involving the tectum, right thalamus, third ventricle, and right lateral ventricle. Severe locoregional mass effect caused brainstem compression and obstructive hydrocephalus (Figure 2). A biopsy was performed with an initial diagnosis of craniopharyngioma. She responded with significant neurologic recovery following shunt placement and was discharged with steroids. The patient subsequently presented to our institution with bilateral lower extremity weakness and gait instability. Neurological examination revealed moon facies and bilateral hip flexor weakness. A brain MRI demonstrated interval tumor progression (Figure 3A,B) and she underwent a craniotomy for tumor debulking of the right thalamic component (Figure 3C,D).

**Figure 4:**
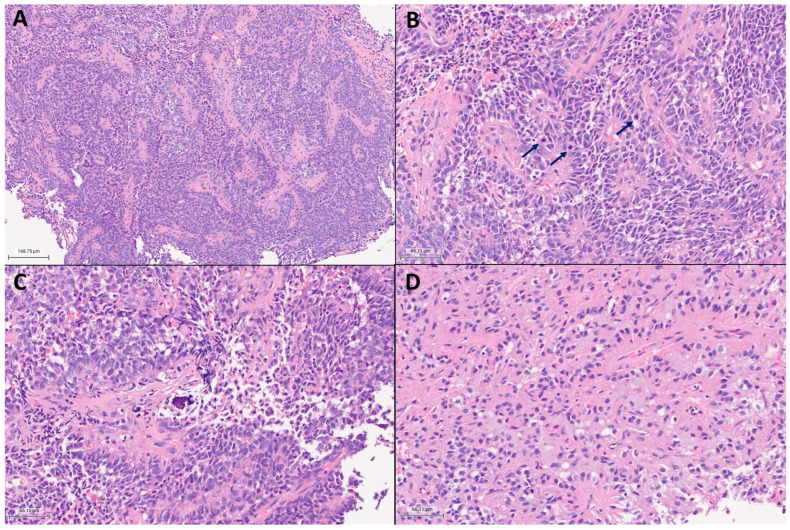
Hematoxylin and eosin staining. (**A**) Ependymoma-like tumor histomorphology with extensive perivascular pseudorosettes. (**B**) Elevated mitotic activity with three mitoses (arrows) in 10 high power fields. (**C**) Focal microcalcifications. (**D**) Microcysts and myxoid change.

**Figure 5:**
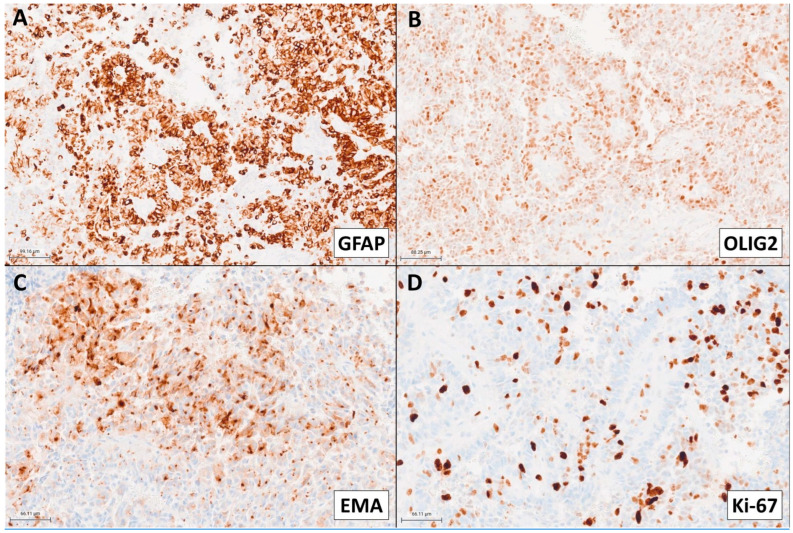
(**A**) Patchy positive GFAP with a subset of fragments demonstrating more prominent expression in perivascular regions. (**B**) Strong to moderate OLIG2 immunopositivity in the majority of tumor nuclei. (**C**) Patchy positive EMA with a strong perinuclear dot-like pattern. (**D**) High Ki-67 (10–30%).

Pathological examination demonstrated a densely cellular neoplasm with elevated mitotic activity and extensive perivascular pseudorosettes (Figure 4). Angulated hyperchromatic nuclei, focal calcifications, and focal myxoid change were present. Necrosis, microvascular proliferation, and oligodendroglioma-like features were not identified within the tissue sections available for histological examination. Olig2 showed strong to moderate immunopositivity in the majority of tumor nuclei (Figure 5B). GFAP was patchy positive with a subset of fragments demonstrating more prominent expression in perivascular regions (Figure 5A). EMA was patchy positive in a strong perinuclear dot-like pattern (Figure 5C). SOX10, synaptophysin, and CAM5.2 were negative. The estimated Ki-67 proliferative index was high (10–30%; Figure 5D). The morphology and intraventricular location bore similarities to supratentorial ependymoma. However, Olig2 positivity in the majority of tumor nuclei raised concern for an alternate diagnosis.

**Figure 6:**
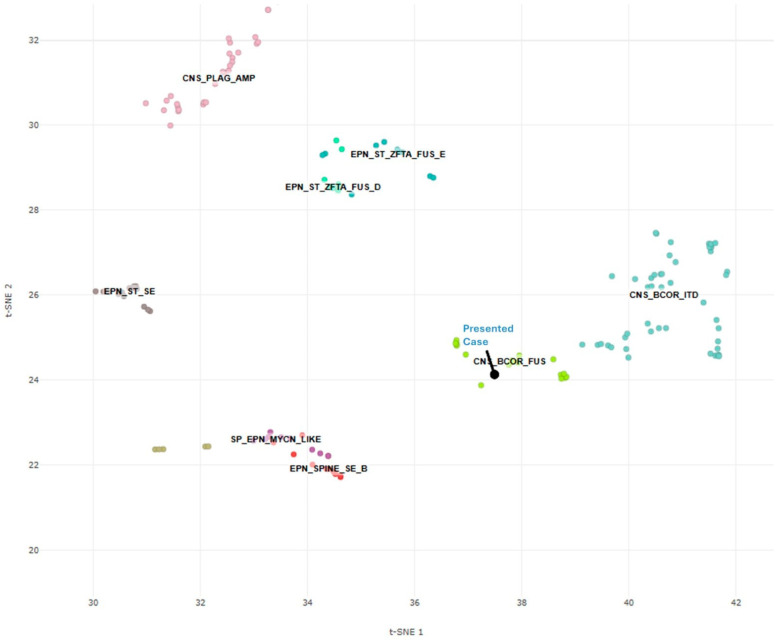
Methylation-based t-SNE distribution. Reference DNA methylation classes (Bethesda CNS tumor classifier v3.0): CNS_BCOR_FUS: CNS tumor with BCOR/BCORL1 fusion; CNS_BCOR_ITD: CNS tumor with BCOR internal tandem duplication; CNS_PLAG_AMP: CNS embryonal tumor with PLAG-family amplification; EPN_ST_SE: subependymoma and ependymoma, supratentorial; EPN_ST_ZFTA_FUS: supratentorial ependymoma, ZFTA fusion-positive; EPN_SPINE_SE: subependymoma and ependymoma, spinal; and SP_EPN_MYCN_LIKE: spinal ependymoma, MYCN-amplified-like.

Further investigation with the Mayo Clinic Neuro-Oncology Expanded Gene Panel with Rearrangement revealed EGFR c.1088C>T (p.T363I) (Exon 9) and c.1793G>T (p.G598V) (Exon 15), clinically relevant variants, as well as a BCOR c.626del (p.S209Cfs*7) (Exon 4) variant. Variants of unknown significance were uncovered, including NOTCH1 c.3076G>A (Exon 19), FUBP1 c.263A>T (Exon 4), and MET c.3272C>T (Exon 15). The fusion assay did not detect a BCOR/BCOR(L1) fusion, prompting further analysis with DNA methylation profiling. The composite methylation profile on the Heidelberg classifier versions 11b6 and 12b6 and the NCI/Bethesda classifier versions 2.0 and 3.0 indicated a consensus match to CNS tumor with BCOR/BCOR(L1) fusion. Dimensionality reduction with UMAP (uniform manifold approximation and projection) and t-SNE (t-distributed stochastic neighbor embedding) also placed the tumor into the same class (Figure 6).

**Figure 7:**
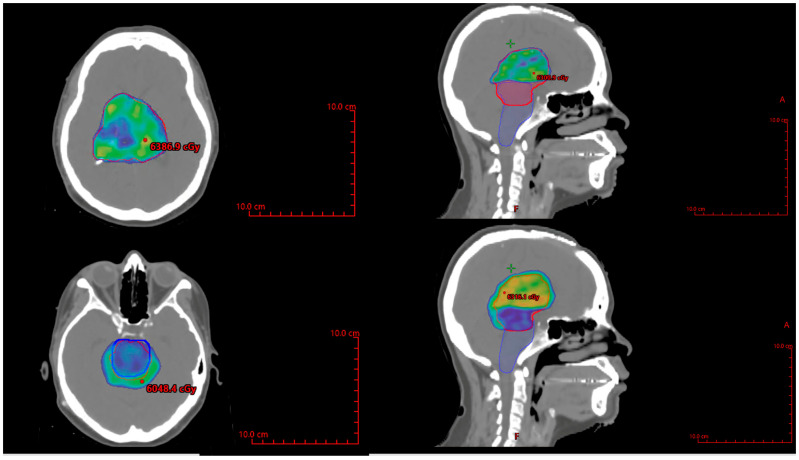
Axial and sagittal views of the radiation treatment plan with PTV shown in red, and brainstem shown in blue, with color wash set to 5940 cGy in the top panels, and 5400 cGy in the bottom panels. PTV = planning treatment volume.

She subsequently developed a bilateral upgaze palsy. Her radiation planning brain MRI showed continued tumor progression (Figure 3E,F). Following a multi-disciplinary discussion coupled with lack of any actionable targets on expression profiling, the decision was made to proceed with external beam radiation therapy (59.4 Gy in 33 fractions) followed by six cycles of adjuvant temozolomide 150–200 mg/m^2^. The planning target volume overlapping the brainstem was significant and was prescribed to receive 54 Gy through dose painting to respect brainstem tolerance (Figure 7). The patient tolerated the treatment well, with improvement in her bilateral upwards gaze palsy and headaches within 2 weeks of starting radiation therapy. She completed radiation treatment with Grade 1 fatigue.

Following three cycles of temozolomide, her MRI brain showed response to treatment with significant tumor reduction (Figure 3G,H). Her symptoms resolved, and she remains alive at 11-month follow-up. Ethical guidelines set out by the Declaration of Helsinki were followed in the preparation of this report, and the patient provided written consent.

## 3. Discussion

The updated WHO classification of CNS tumors improves upon the prior classification schemas by incorporating histologic, molecular, and epigenetic characteristics into an integrated diagnosis [22]. DNA methylation profiling has emerged as an effective means to identify new CNS tumor entities with distinct genetic and epigenetic features within the CNS-PNET group. BCOR-ITD is a newly identified tumor among CNS-PNETs, wherein a match to this methylation class on DNA methylation profiling is an essential criterion for unresolved cases.

BCOR alterations occur in a variety of tumors, including CNS tumors with BCOR/BCOR(L1) fusion, CNS tumors with BCOR-ITD, gliomas, medulloblastomas, and specific sarcomas [3,14,17,23]. CNS tumors with BCOR/BCOR(L1) fusion are characterized by the presence of a BCOR fusion, often paired with EP300 as the typical fusion partner, with or without hallmark CNV within chromosomes 22q12.31 and Xp11.4 [17]. However, CNS tumors without BCOR/BCOR(L1) fusion that instead harbor copy number variations within chromosome Xp11.4 and 22q12.31 or truncating small nucleotide variants can match to the same methylation class as those with BCOR/BCOR(L1) fusion [14,17]. To our knowledge, this is the third reported case of a CNS tumor with BCOR/BCOR(L1) fusion matching the methylation class with a BCOR small nucleotide variant (BCOR c.626del (p.S209Cfs*7) (Exon 4)) instead of a fusion. These cases are exceptionally rare, but share similar molecular characteristics [14]. BCOR deletions hinder PRC1.1 tumor suppressor functioning and incomplete PRC1.1 dysfunction may be sufficiently adequate to promote tumorigenesis [10,24].

CNS tumors with BCOR/BCOR(L1) fusion predominantly occur in adults [14,18,19], consistent with the age of our patient at diagnosis of 37 years. In keeping with the reported radiographic features of CNS tumors with BCOR/BCOR(L1) fusion and predilection for the ventricular region, our patient’s tumor involved the lateral ventricle and demonstrated T1 hypointensity and T2 hyperintensity [17]. These tumors can appear well-demarcated or diffusely infiltrating and with or without enhancement [17,20]. Our patient’s tumor was well-demarcated, enhancing, diffusion-restricting, partially calcified, and partially cystic.

Collectively, the histomorphologic examination of our patient’s tumor corroborates other reported cases of CNS tumors with BCOR/BCOR(L1) fusion, which exhibit diverse histologic findings including oligodendroglial-like chicken-wire vasculature and/or ependymomal-like perivascular pseudorosettes [17,19]. With the exception of supratentorial ZFTA fusion-positive ependymomas which can show patchy Olig2 positivity, extensive Olig2 and patchy GFAP positivity are otherwise atypical in ependymomas [19] and should prompt further consideration for an alternative diagnosis such as CNS tumors with BCOR/BCOR(L1) fusion, as substantiated in our case. Malignant features, including necrosis and elevated mitotic activity are frequently observed in these tumors [19]. Microvascular proliferation is less commonly demonstrated [19]. Our patient’s tumor was highly proliferative with frequent mitotic activity and an estimated Ki-67 ranging from 10–30%. Notably, EMA showed focal strong dot-like perinuclear immunoreactivity, which has not been previously reported in these tumors.

Our patient’s tumor also harbored EGFR p.G598V and p.T363I pathologic variants. In glioblastoma, EGFR p.T363I has been reported in cases of glioblastoma in a single tumor sector [25]. In contrast, EGFR p.G598V is one of the most common extracellular domain missense mutations [26]. EGFR p.G598V is not known to be associated with survival [26]. However, preclinical data suggest that glioblastoma with EGFR missense mutants may be responsive to EGFR kinase inhibitors [27,28,29]. The presence of these EGFR missense mutations in our patient’s tumor may be therapeutically relevant for second-line therapy in the setting of recurrence [26,28]. However, treatment resistance [29] has also been demonstrated in certain cases [28].

In view of the limited evidence on therapies for this extremely rare tumor that matched the methylation class of CNS tumors with BCOR/BCORL1 fusion without a detectable fusion, we reasoned that radiation therapy was warranted given the high proliferative activity. Temozolomide was also selected for its efficacy in CNS tumors with BCOR/BCORL1 fusion and its favorable toxicity profile [16,30,31]. Bevacizumab, carboplatin, ifosfamide, cisplatin, and etoposide also have proven efficacy, but carry greater risks of toxicities [16,19,30] (Table 1).

We acknowledge that several limitations exist in this study. The nature of a single case may not reflect the broader population of CNS tumors with BCOR/BCORL1 fusions in their entirety or those tumors matching to the same methylation class. The short follow-up in our patient limits the understanding of the durability of this treatment regimen and the sustainability of the response to therapy.

Future investigations should be directed at deepening our understanding of the clinical and biological behavior of these tumors. Evidence suggests that these tumors are unique and distinguishable from CNS tumors with BCOR-ITD. Therapeutics represent an open area of investigation, and forthcoming studies should be directed at identifying effective therapies for these tumors. As we cultivate our understanding of CNS tumors with BCOR/BCORL1 fusions, the management for these patients will also continue evolve.

Our case adds to the limited but growing body of evidence for a new CNS tumor type or subtype that may be incorporated into the future WHO classification of CNS tumors. Substantial evidence, including therapeutic options, remains to be uncovered for CNS tumors with BCOR(L1) fusions.

## 4. Conclusions

A CNS tumor with BCOR/BCOR(L1) fusion is a rare tumor entity, not yet defined in the 2021 WHO classification of CNS tumors. Our case adds to the growing body of evidence for definitive radiation and temozolomide for the treatment of this rare tumor entity.

## Figures and Tables

**Table 1:** CNS Tumor with BCOR fusion treatments.

PMID	Age	Sex	Location	Fusion	Initial Diagnosis	Treatment	PFS (Months)	OS (Months)
Pisapia et al. [30]	15	M	right frontal, left temporal, left occipital	BCOR:CREBBP	diffuse astrocytic glioma, with molecular features of glioblastoma	RT with concomitant TMZ	18	27
Baressi et al. [18]	45	M	right frontal	CREBBP:BCORL1	-	RT and TMZ	1	1
Yamazaki et al. [16]	17	F	right frontal	CREBBP:BCORL1	-	resection	18	-
Tauziède-Espariat et al. [20]	13	M	right temporal	EP300:BCOR	-	GTR and chemotherapy	16	16
Tauziède-Espariat et al. [20]	27	M	left frontal	EP300:BCOR	-	GTR, chemotherapy and focal RT	27	27
Tauziède-Espariat et al. [19]	64	M	left frontal	EP300:BCOR	high-grade glioneuronal tumor	GTR	12	12
Tauziède-Espariat et al. [19]	40	M	within the right lateral ventricle	EP300:BCOR	high-grade glioneuronal tumor	STR, carboplatin and VP16	12	12
Torre et al. [21]	12	M	right frontal	EP300:BCOR	oligodendroglioma	extraventricular drainage, STR	4.5	6
Torre et al. [21]	10	F	left basal ganglia and thalamus	EP300:BCOR	glioblastoma	STR	1	7
Torre et al. [21]	18	M	right medial occipital	EP300:BCOR	dysembryoplastic neuroepithelial tumor (DNET) versus glioneuronal tumor	STR	15	42
Fukuoka et al. [32]	72	M	occipital	EP300:BCORL1	anaplastic ependymoma G3	resection, RT (40 Gy), chemotherapy	24	33
Xu et al. [31]	43	F	right frontotemporal	EP300:BCOR	-	NTR, RT (5200 cGy), adjuvant chemotherapy	8	8
Xu et al. [31]	54	M	right frontal	EP300:BCOR; BCOR-L3MBTL2	-	GTR, RT (6000 cGy) and adjuvant TMZ	16	16
Yamazaki et al. [16]	18	F	right frontal	-	-	resection	33	-
Yamazaki et al. [16]	21	F	right frontal	-	-	resection, RT (54 Gy), TMZ	89	-
Yamazaki et al. [16]	25	F	right frontal	-	-	resection, SRS, adjuvant chemotherapy with bevacizumab, surgery, later addition of ifosfamide, cisplatin, and etoposide	-	115

RT = radiation therapy; TMZ = temozolomide; GTR = gross total resection; STR = subtotal resection; and SRS = stereotactic radiosurgery.

## Data Availability

The data presented in this study are available on request from the corresponding author due to privacy and ethical restrictions.

## Abbreviations

The following abbreviations are used in this manuscript:

CNS	central nervous system tumor
BCOR	BCL6 corepressor gene
CT	computed tomography
GTR	gross total resection
ITD	internal tandem duplication
MRI	magnetic resonance imaging
RT	radiation therapy
SRS	stereotactic radiosurgery
STR	subtotal resection
t-SNE	t-distributed stochastic neighbor embedding
TMZ	temozolomide
UMAP	uniform manifold approximation and projection
WHO	World Health Organization
